# Forkhead Transcription Factor FOXO3a Levels Are Increased in Huntington Disease Because of Overactivated Positive Autofeedback Loop*

**DOI:** 10.1074/jbc.M114.612424

**Published:** 2014-09-30

**Authors:** Kaja Kannike, Mari Sepp, Chiara Zuccato, Elena Cattaneo, Tõnis Timmusk

**Affiliations:** From the ‡ Department of Gene Technology, Tallinn University of Technology, Tallinn 12618, Estonia and; the § Department of Pharmacological Sciences and Center for Stem Cell Research, University of Milan, Milano 20133, Italy

**Keywords:** Akt PKB, Chromatin Immunoprecipitation (ChIP), FOXO, Huntington Disease, Nuclear Translocation, Autoregulation, 3-Nitropropionic Acid (3-NP), Hdh Cell Line, Neurons, R6/2 Mice, Post-mortem Brain Tissue

## Abstract

Huntington disease (HD) is a fatal autosomal dominant neurodegenerative disorder caused by an increased number of CAG repeats in the *HTT* gene coding for huntingtin. Decreased neurotrophic support and increased mitochondrial and excitotoxic stress have been reported in HD striatal and cortical neurons. The members of the class O forkhead (FOXO) transcription factor family, including FOXO3a, act as sensor proteins that are activated upon decreased survival signals and/or increased cellular stress. Using immunocytochemical screening in mouse striatal Hdh^7/7^ (wild type), Hdh^7/109^ (heterozygous for HD mutation), and Hdh^109/109^ (homozygous for HD mutation) cells, we identified FOXO3a as a differentially regulated transcription factor in HD. We report increased nuclear FOXO3a levels in mutant Hdh cells. Additionally, we show that treatment with mitochondrial toxin 3-nitropropionic acid results in enhanced nuclear localization of FOXO3a in wild type Hdh^7/7^ cells and in rat primary cortical neurons. Furthermore, mRNA levels of *Foxo3a* are increased in mutant Hdh cells compared with wild type cells and in 3-nitropropionic acid-treated primary neurons compared with untreated neurons. A similar increase was observed in the cortex of R6/2 mice and HD patient post-mortem caudate tissue compared with controls. Using chromatin immunoprecipitation and reporter assays, we demonstrate that FOXO3a regulates its own transcription by binding to the conserved response element in *Foxo3a* promoter. Altogether, the findings of this study suggest that FOXO3a levels are increased in HD cells as a result of overactive positive feedback loop.

## Teaser

**Background:** Forkhead box O (FOXO) transcription factors, integrators of stress and survival signals, have been implicated in neurodegenerative diseases including Huntington disease (HD).

**Results:** FOXO3a regulates its own transcription and its levels are increased in HD.

**Conclusion:** Positive autofeedback loop contributes to elevated FOXO3a activity in HD.

**Significance:** Autoregulation of FOXO3a adds complexity to transcription dysregulation in HD.

## Introduction

Huntington disease (HD)^2^ is a neurodegenerative disorder that manifests in chorea, psychiatric disturbances, and cognitive decline. Genetically, HD is an autosomal dominant disease caused by a CAG repeat expansion in the *HTT* (*huntingtin*) gene, which translates into a polyglutamine tract in HTT protein (1). Neuropathologically, HD is characterized by loss of medium sized spiny neurons in the striatum and pyramidal neurons in the cerebral cortex (2). Several pathogenic mechanisms have been described that mediate neuronal dysfunction and death; these mechanisms include transcriptional dysregulation, loss of neurotrophic support, mitochondrial impairment, cellular stress, and excitotoxicity (3, 4). A number of transcriptional pathways are disrupted in HD including decreased expression of genes involved in growth factor, neurotransmitter and calcium signaling, and up-regulation of some genes associated with cellular stress (5). Notably, reduced brain-derived neurotrophic factor (BDNF) expression and its delivery to striatal targets in addition to decreased levels of TrkB receptors have been described in HD (6–9). Mitochondrial dysfunction hypothesis is supported by the fact that 3-nitropropionic acid (3-NP), an irreversible inhibitor of mitochondrial complex II, induces striatal degeneration similar to HD in rodents and primates (10–12). Additionally, mutant huntingtin disrupts mitochondrial Ca^2+^ homeostasis, promotes formation of reactive oxygen species, and sensitizes cells to excitotoxic stimuli and apoptosis (13–15). The relative importance of different disease mechanisms is unclear, but overall imbalance between activation of prosurvival and apoptotic pathways has been suggested.

Forkhead box O (FOXO) family proteins FOXO1, FOXO3a, FOXO4, and FOXO6 act as cellular sensors of stress and survival signals. They regulate transcriptional programs that affect differentiation, survival, longevity, cell cycle, metabolism, stress resistance, autophagy, and tumor suppression (16). The activity of FOXO proteins is precisely controlled by post-translational modifications, mainly phosphorylation, acetylation, ubiquitylation, and methylation (17–19). Growth factor-induced phosphorylation of FOXOs by AKT/PKB (protein kinase b) leads to inactivation and redistribution of FOXOs from nucleus to cytosol, a mechanism mediating also the neuroprotective signaling of BDNF (20–23). Another pathway activated by BDNF is Ras/MAPK/ERK1/2, which results in phosphorylation of FOXO3a and directs it to proteasomal degradation (24). *FOXO* mRNAs are widely expressed at varying levels in mammalian tissues; compared with *Foxo1* and *Foxo4*, *Foxo3a* displays the highest expression in the brain (25, 26).

Here we screened for transcription factors dysregulated in HD, using a panel of over 200 antibodies. One of the transcription factors identified was FOXO3a. We examined localization, expression, and regulation of FOXO3a using different HD models: striatal cell lines from mutant *Htt* knock-in mice, 3-NP-treated rat primary cortical neurons, and R6/2 transgenic mice. Additionally, we analyzed mRNA levels of *FOXO3a* in post-mortem caudate and cerebral cortex of HD patients. Our results suggest that activity of FOXO3a is increased in HD models and in HD patients through mechanisms involving positive autoregulation.

## MATERIALS AND METHODS

### 

#### 

##### Human Samples

Post-mortem human brain tissues were obtained from the Harvard Brain Tissue Resource Center. Cortex tissues were from controls 5074, 5936, 5959, 08704, and 13574 and from HD patients 5570, 6121, 0497, 0950, and 18590. Caudate nucleus tissues were from controls 5936, 5959, and 6142 and from HD patients 5507, 6010, and 6183. Distribution by disease grade is as follows: HD grade 2: 6051 and 6121; HD grade 3: 0950, 5570, 6010, 6183, and 18590; and HD grade 4: 0497 and 5507. All diagnoses were based on clinical assessment and histopathological evaluation by experienced neuropathologists according to Vonsattel classification. The use of these tissues has been approved by the Università degli Studi Milano ethical board following the guidelines of the Declaration of Helsinki.

##### Animal Procedures

All animal procedures were performed in compliance with the local ethics committee. The R6/2 and control mice were housed using a normal light/dark cycle. After overnight starvation, the 6-week-old animals were sacrificed and dissected to separate the different neuronal areas. Sprague-Dawley rats were mated, and females were sacrificed in a CO_2_ chamber on day 23 of gestation for isolation of the fetuses.

##### Constructs

pFLAG-FOXO3A-WT (Addgene plasmid no. 8360) and pFLAG-FOXO3A-TM (Addgene plasmid no. 8361) have been described previously (27). For pEGFP-FOXO3A construct, the KspAI and EcoRI fragment of pFLAG-FOXO3A-WT containing the entire FOXO3A coding sequence was cloned into pEGFP-C1 vector (Clontech). For *Foxo3a* promoter constructs FL, Δ4, Δ3–4, Δ1–4, FLmut3 mouse genomic DNA regions chr10:41996473–41998267, 41996471–41998135, 41996471–41997927, and 41996471–41997399 (according to mouse genome assembly NCBI37/mm9) were PCR-amplified and inserted into pGL4.15[luc2P/Hygro] vector (Promega). For pGL4.83[hRlucP/PGK1/Puro] mouse 3-phosphoglycerate kinase 1 (*PGK1*) promoter sequence (ChrX: 103382066–103382573 according to NCBI37/mm9 genome assembly) was inserted into pGL4.83[hRlucP/Puro] (Promega). The *Renilla* luciferase encoding vector with *EF1*α promoter pGL4.83[hRlucP/EF1α/Puro] has been described previously (28).

*In silico* analysis of potential FHREs in *Foxo3a* promoter sequence was performed using MatInspector software (Genomatix). For site-directed mutagenesis of FHRE in region 3 of the *Foxo3a* FL promoter construct, complementary primers against the target sequence containing the respective mutation (5′-CACACACGTGTGCTGGgtACAAGCGCGCCAG-3′) and Phusion high fidelity DNA polymerase (Thermo Scientific) were used.

##### Cell Culture and Transfections

The conditionally immortalized striatal progenitor Hdh^7/7^, Hdh^7/109^, and Hdh^109/109^ cells have been described previously (29). Briefly, these cells are derived from primary striatal cells from mice with different *Htt* genotypes and immortalized with temperature-sensitive large T antigen. Hdh^7/7^ cells are from wild type mice carrying two copies of the endogenous *Htt* allele with 7 CAG repeats; Hdh^7/109^ are from heterozygous, and Hdh^109/109^ are from homozygous knock-in mice with one or both *Htt* alleles having 109 CAG repeats, respectively. Hdh cells were propagated in DMEM (Invitrogen) supplemented with 10% fetal bovine serum (PAA Laboratories), 100 units/ml penicillin, and 0.1 mg/ml streptomycin (PAA Laboratories) at 33 °C in 5% CO_2_. Hdh cells cultured on 48-well plates were transfected using Lipofectamine 2000 (Invitrogen) at reagent:DNA ratio 2:1. For luciferase assays, 0.125 μg of effector protein construct, 0.125 μg of firefly luciferase construct, and 10 ng of *Renilla* luciferase construct pGL4.83[hRlucP/PGK1/Puro] were used. When indicated, Hdh^7/7^ cells were treated with 1 mm 3-NP (Sigma-Aldrich) for 48 h.

HEK293 cells were propagated in MEM (Invitrogen) supplemented with 10% fetal bovine serum (PAA), 100 units/ml penicillin, and 0.1 mg/ml streptomycin (PAA). LipoD293 (Signagen) and RNAiMAX (Invitrogen) were utilized for plasmid and siRNA transfections, respectively, in HEK293 cells according to the manufacturers' protocols. Predesigned Silencer Select siRNAs against FOXO3a s80658 (siRNA1) and s1408638 (siRNA2) and negative control 1 siRNA (scrambled) were purchased from Ambion.

For rat cortical neuronal cultures the cortical hemispheres from embryonic day 22.5 embryos of Sprague-Dawley rats were dissected, and the underlying diencephalons, hippocampi, and striata were trimmed away. The obtained cortices were treated as described previously (30). Neuronal cultures were used for analyses at 6–8 days *in vitro*. Neurons cultured on 48-well plates were transfected at days 5 and 6 *in vitro* using Lipofectamine 2000 (Invitrogen) at reagent:DNA ratio 2:1. For cytochemical analysis, 0.5 μg of pEGFP-FOXO3A-WT encoding vector was used, and for luciferase assays, 0.25 μg of effector protein construct, 0.25 μg of firefly luciferase construct, and 10 ng of *Renilla* luciferase construct pGL4.83[hRlucP/EF1α/Puro] were used. When indicated, neurons were treated with 0.5 mm 3-NP for 2–24 h.

##### Immunocytochemistry

Cells grown on poly-l-lysine-coated coverslips were fixed in 4% paraformaldehyde in PBS for 15 min, treated with 50 mm NH_4_Cl in PBS for 10 min, permeabilized in 0.5% Triton X-100 in PBS for 15 min, and blocked with 2% bovine serum albumin in PBS. Incubations with rabbit polyclonal anti-FOXO3a 1:200 (1112; Cemines, epitope SADDSPSQLSKWPGS) and Alexa 488- or Alexa 546-conjugated goat anti-rabbit IgG 1:2000 (Molecular Probes) antibodies were carried out in 0.2% BSA and 0.1% Tween 20 in PBS at room temperature for 1.5 h each. The samples were mounted in ProLong Gold antifade reagent with 4′-6-diamidino-2-phenylindole (Molecular Probes) and analyzed by confocal microscopy (LSM Duo; Zeiss).

##### Western Blotting

Cells were lysed in radioimmune precipitation assay buffer (50 mm Tris-HCl, pH 8, 150 mm NaCl, 1% Nonidet P-40, 0.5% sodium deoxycholate, 0.1% SDS, 1 mm dithiothreitol, and protease inhibitors mixture Complete mini (Roche)); if necessary, phosphatase inhibitors mixture (Roche) was added. Cell lysates were sonicated for 15 s with 30% amplitude on Sonics VibraCell and centrifuged at 16,100 × *g* for 15 min at 4 °C. Nuclear and cytosolic fractions were prepared as described previously (29). Protein concentrations in lysates were measured with BCA assay (Pierce). Equal amounts of protein were separated in 8% SDS-PAGE and transferred to PVDF membrane (Bio-Rad). Membranes were blocked overnight at 4 °C in 5% skim milk and 0.1% Tween 20, incubated with primary antibodies and secondary antibodies in 0.1% Tween 20 with 2% skim milk in PBS or 5% BSA in TBS (in case of phospho-site specific antibodies). Antibodies were diluted as follows: rabbit polyclonal anti-FOXO3a 1:1000 (1112; CeMines), rabbit polyclonal anti-Flag 1:1000 (F7425; Sigma-Aldrich), mouse monoclonal anti-tubulin β to a final concentration of 30 ng/ml (E-7; Developmental Studies Hybridoma Bank), rabbit polyclonal anti-HDAC2 1:1000 (sc-7899; Santa Cruz), rabbit polyclonal anti-pFOXO3a(Ser-253) 1:1000 (06–953; Upstate/Millipore), rabbit polyclonal anti-pFOXO3a(Ser-294) 1:1000 (A16693; Life Technologies), rabbit polyclonal anti-AKT1/2/3 1:2000 (sc-8312; Santa Cruz), rabbit polyclonal anti-pAKT(Ser-473) 1:2000 (9271; Cell Signaling), mouse monoclonal anti-ERK1/2 1:1000 (sc-135900; Santa Cruz), mouse monoclonal anti-pERK 1:1000 (sc-7383; Santa Cruz), mouse monoclonal anti-GAPDH 1:2000 (MAB374; Chemicon), and HRP-conjugated goat anti-mouse/rabbit IgG 1:2500 (Thermo Scientific). Chemoluminescent signal was detected using SuperSignal West Femto chemoluminescent substrate (Thermo Scientific) and ImageQuant 400 imaging system (Amersham Biosciences). Images were quantified with ImageQuant T4 v2005 software (Amersham Biosciences).

##### RNA Extraction and Reverse Transcription

Total RNAs from 200–300 mg of human cortex and/or striatum tissue of samples 5074, 5936, 5959, 6142, 6051, 6121, 5570, 6010, 6183, and 5507 were isolated by using 2 ml of TRIzol reagent (Invitrogen), after the tissues had been homogenized in liquid nitrogen with a mortar and pestle. The concentration of RNA was evaluated spectrophotometrically, and its quality was verified by means of agarose gel electrophoresis of 1 μg of each sample. Genomic DNA was digested using 1 unit of rDNase I (Applied Biosystems) per 1 μg/ml of total RNA at 37 °C for 10 min following the manufacturer's instructions. From human cortex tissue samples 08704, 13574, 00497, 00950, and 18590, the total RNAs were extracted from 50 mg of tissue using RNeasy lipid tissue mini kit (Qiagen) following the manufacturer's instructions. The RNA integrity number and concentrations were assessed by a RNA 6000 Nano Kit (Agilent) according to the manufacturer's instructions; all samples used had RNA integrity numbers above 6.

Total RNA from R6/2 mice tissue was extracted by lipid tissue mini kits (Qiagen) from 100 mg of tissue and treated with 2 units of Turbo DNase (Invitrogen) following the manufacturer's instructions. Total RNAs from Hdh cells and primary cortical neurons were extracted using a RNeasy micro kit (Qiagen) following the manufacturer's instructions. First strand cDNAs were synthesized from 0.3–5 μg of total RNA with Superscript III first strand synthesis system (Invitrogen) with oligo(dT) or with combination of oligo(dT) and random decamer primers.

##### Chromatin Immunoprecipitation

Cells on 100-mm dishes were cross-linked in 1% formaldehyde, 100 mm NaCl, 0.5 mm EGTA, 50 mm HEPES (pH 8.0) for 10 min at room temperature. One-tenth volume of 1.25 m glycine was added for quenching, and cells were washed twice with PBS and lysed in buffer containing 1% SDS, 10 mm EDTA, 50 mm Tris-HCl (pH 8.0) and protease inhibitors cocktail (Roche). Lysates were sonicated five times for 5 s at 50% amplitude on Sonics VibraCell to obtain 200–1000-bp fragments of genomic DNA, and insoluble material was spun down for 5 min at maximum speed in a tabletop centrifuge at 4 °C. 400 μg of the primary neuron lysates or 1000 μg of Hdh^7/7^ and Hdh^109/109^ cell lysates were diluted 1:9 with dilution buffer (1% Triton X-100, 150 mm NaCl, 2 mm EDTA, 20 mm Tris-HCl, pH 8.0, protease inhibitors cocktail (Roche)) and incubated with 5 μg FOXO3a antibodies (1112; Cemines), acetyl-histone H4 antibodies (06-866; Millipore) or without antibodies overnight at 4 °C. 60 μl of 50% protein A-Sepharose slurry (GE Healthcare), preabsorbed in 200 μg/ml BSA and 10 μg/ml sheared salmon sperm DNA, was added per reaction for 6–8 h. Sepharose-chromatin complexes were washed 3 times with wash buffer (1% Triton X-100, 0.1% SDS, 150 mm NaCl, 2 mm EDTA, 20 mm Tris-HCl (pH 8.0), protease inhibitors cocktail (Roche)) and once with wash buffer containing 500 mm NaCl. The immune complexes were eluted two times by addition of 75 μl of elution buffer (1% SDS, 100 mm NaHCO_3_, 1 mm EDTA), and the eluates of the same sample were combined. Cross-links were reversed by incubating the eluates in 200 mm NaCl at 65 °C overnight. DNA was purified with QIAquick PCR purification kit (Qiagen): 500 μl of QIAquick binding buffer, 100 μl of water, and sodium acetate to 60 mm concentration were added to the eluates, and the mixtures were subjected to columns as described in the manufacturer's protocol. DNA was eluted with 50 μl of 10 mm Tris-Cl (pH 8.5).

##### Quantitative PCR

LightCycler 2.0 engine (Roche), qPCR Core kit for SYBR® Green I No ROX (Eurogentec) and polycarbonate qPCR capillaries (Bioron GmbH) were used to perform quantitative PCR. The reactions were carried out in triplicate in a volume of 10 μl containing 1/80 of reverse transcription reaction or 1/50 of DNA from chromatin immunoprecipitation eluate. The following cycling conditions were used: 95 °C for 5 min, 45 cycles of 95 °C for 10–15 s, 55–62 °C for 10–20 s, and 72 °C for 10–20 s. Melting curve analysis was performed at the end of each reaction to confirm amplification of a single PCR product. The primers for expression analysis of mouse *Foxo3a* were 5′-TACGAGTGGATGGTGCGCTG-3′ and 5′-AGGTTGTGCCGGATGGAGTTC-3′; rat *Foxo3a* 5′-TACGAGTGGATGGTGCGCTG-3′ and 5′-AGGTTGTGGCGGATGGAGTTC-3′; and human *FOXO3a* 5′-TTCAAGGATAAGGGCGACAGCAAC-3′ and 5′-CTGCCAGGCCACTTGGAGAG-3′. Primers for mouse and rat *FasL* were 5′-AAGGAACTGGCAGAACTCCGTG-3′ and 5′-GTTGCAAGACTGACCCCGGAAG-3′. Target gene mRNA expression levels in mouse and rat were normalized to the levels of hypoxanthine-guanine phosphoribosyltransferase (*HPRT1*) transcripts detected with primers 5′-CAGTCCCAGCGTCGTGATTA-3′ and 5′-AGCAAGTCTTTCAGTCCTGTC-3′. Results from human HD post-mortem material were normalized to geometric mean of mRNA levels of three normalizing genes: *HPRT1*, 5′-GCCAGACTTTGTTGGATTTG-3′ and 5′-CTCTCATCTTAGGCTTTGTATTTTG-3′; *SDHA* (succinate dehydrogenase complex, subunit A), 5′-TGGGAACAAGAGGGCATCTG-3′ and 5′-CCACCACTGCATCAAATTCATG-3′; and *HMBS* (hydroxymethylbilane synthase), 5′-GGCAATGCGGCTGCAA-3′ and 5′-GGGTACCCACGCGAATCAC-3′. The primers used were 5′-TCCTTTCCCTCCTCCCTGC-3′ and 5′-ACGCCTCTCGCTCCTCTT-3′ for mouse *Foxo3a* promoter and 5′-CCAGCCTCACATTCCATTTC-3′ and 5′-GCGCTTGTTTACCAGCACAC-3′ for rat *Foxo3a* promoter. Primers against unrelated region for mouse were 5′-GTGGCTATGTGGTGTTTCAGGT-3′ and 5′-TGTGGGAGCAGAGAAGCCTA-3′ and for rat 5′-TAGACCCAGGAGGGAGTTATTTAAGAG-3′ and 5′-TTGGGAATGCAATGCAGTGTGTAC-3′.

##### Luciferase Assay

48 h post-transfection cells on 48-well plates were lysed in 50 μl of passive lysis buffer (Promega), and reporter activities were measured using Dual-Glo luciferase assay (Promega) and GENios Pro multifunction microplate reader (Tecan). The reactions were carried out in duplicate. Background signals from untransfected cells were subtracted, and firefly luciferase signal values were normalized to *Renilla* luciferase signals.

##### Statistics

qPCR data were analyzed essentially as described previously (31). Briefly, data were log-transformed and autoscaled, means and S.D. values were calculated, and two-tailed paired *t* tests were performed. In case of analysis of mRNA levels in post-mortem human tissues, two-tailed equal variance *t* tests were used. The data were back-transformed into the original scale for graphical depiction. The *error bars* represent upper and lower limits backtransformed as mean + S.D. and mean − S.D., respectively. Western blot and luciferase assay data were log-transformed to ensure normal distribution, means and S.D. values were calculated, and two-tailed paired *t* tests and unpaired *t* tests were used as appropriate. The numbers of experiments are indicated under “Results.”

## RESULTS

### 

#### 

##### Immunocytochemical Screening Reveals Differential FOXO3a Signal in Hdh Cells

**FIGURE 1.**
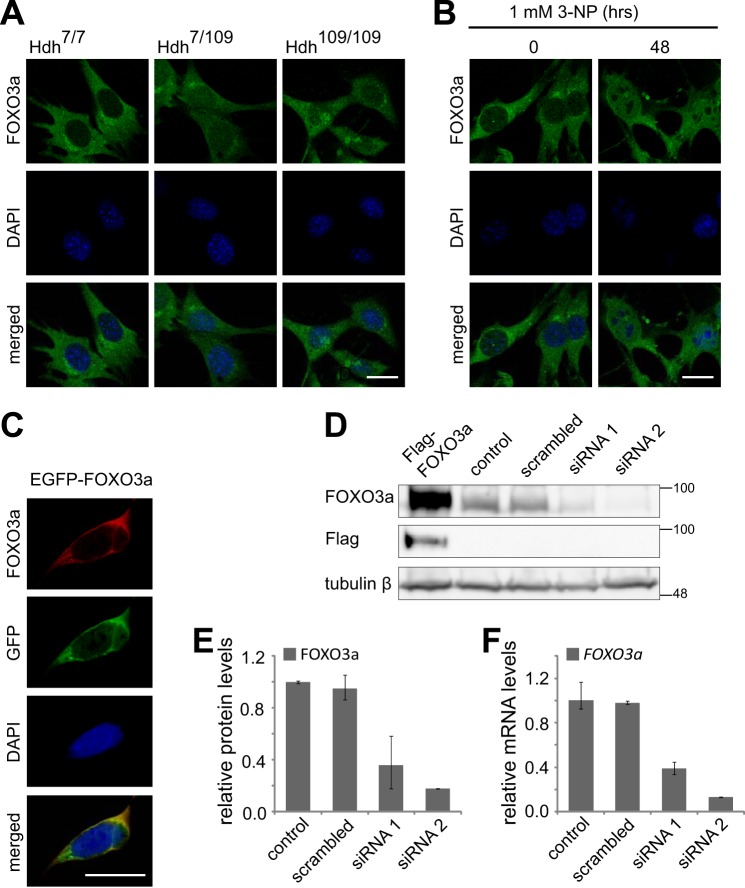
**Localization of FOXO3a-like immunoreactive signal in Hdh cells and validation of FOXO3a antibody.** *A*, representative micrographs demonstrating differential distribution of FOXO3a antibody staining in Hdh^7/7^, Hdh^7/109^, and Hdh^109/109^ cells. *B*, immunocytochemical analysis with FOXO3a antibodies in Hdh^7/7^ cells left untreated or treated with 1 mm 3-NP for 48 h. *C*, co-localization of EGFP signal and FOXO3a antibody staining in HEK293 cells transfected with EGFP-FOXO3a encoding construct. In *A–C*, DNA was counterstained with DAPI. *Scale bar*, 20 μm. *D*, Western blot analysis with FOXO3a antibodies of HEK293 cells left untransfected (control) or transfected with Flag-FOXO3a encoding construct, scrambled siRNA, or siRNAs 1 and 2 against *FOXO3a*. Flag-specific antibodies were used for verification of Flag-FOXO3a expression and tubulin β served as loading control. *E*, quantification of the data in *D*. FOXO3a signals were normalized to tubulin β signals. *F*, RT-qPCR analysis of *FOXO3a* mRNA levels in siRNAs transfected HEK293 cells performed in parallel with Western blotting. In *E* and *F*, the mean results from two independent experiments are shown.

The hypothesis of transcriptional dysregulation in HD is reinforced by several studies demonstrating pathological alterations in nuclear translocation of transcription factors. For example, subcellular distribution of neuron-restrictive silencer factor (REST/NRSF) and sterol regulatory element-binding protein (SREBP) has been demonstrated to be changed in mutant huntingtin-expressing cells (29, 32). To identify mislocalized transcription factors in HD, we immunostained mouse striatal Hdh^7/7^ (wild type), Hdh^7/109^ (heterozygous for HD mutation), and Hdh^109/109^ (homozygous for HD mutation) cells with a panel of 200 antibodies against various transcription factors. One of the antibodies identified by the screening was an antibody generated against a peptide in FOXO3a. As shown in Fig. 1*A* FOXO3a-like immunoreactive signal in Hdh^7/7^ cells was mainly cytoplasmic, whereas in heterozygous and homozygous mutant Hdh cells, the signal was detected also in the nucleus. To validate this observation in a different cell stress condition, we tested whether 3-NP-induced mitochondrial stress, widely used to mimic HD in rodents and in non-human primates (10, 33), is able to influence the distribution of FOXO3a-like immunoreactive signal in Hdh^7/7^ cells. As demonstrated in Fig. 1*B*, treatment with 1 mm 3-NP for 48 h considerably increased the amount of FOXO3a-like immunoreactive signal in the nuclei of Hdh^7/7^ cells. Thus, mitochondrial toxin 3-NP induced Hdh^109/109^-like nuclear staining with FOXO3a antibodies in Hdh^7/7^ cells.

To determine the target specificity of the FOXO3a antibodies used, we transfected HEK293 cells with constructs encoding EGFP- or Flag-tagged FOXO3a and with scrambled or *FOXO3a*-specific siRNAs. By cytochemistry we observed co-localization of signals visualized by direct EGFP fluorescence or by indirect immunolabeling with FOXO3a antibodies in cells expressing EGFP-FOXO3a fusion protein (Fig. 1*C*). Western blotting revealed that FOXO3a antibodies recognized overexpressed Flag-FOXO3a protein, as well as an endogenous protein of similar molecular mass (Fig. 1*D*). Compared with untransfected control or scrambled siRNA transfected cells, the intensity of the endogenous signal obtained by immunoblotting with FOXO3a antibodies was reduced to 39 or 13% in cells transfected with *FOXO3a*-specific siRNA 1 or 2, respectively (Fig. 1, *D* and *E*). Similar reduction was seen in *FOXO3a* mRNA levels by RT-PCR analysis performed in parallel (Fig. 1*F*). Collectively, the above results verify the specificity of the antibodies and suggest that the control of FOXO3a localization is disturbed in HD cells.

##### FOXO3a Protein Levels Are Elevated in Mutant Hdh Cells

**FIGURE 2.**
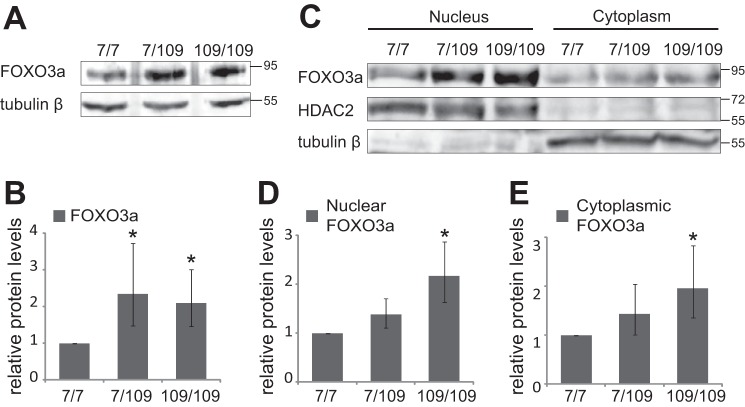
**Levels of FOXO3a protein in Hdh cells.** *A*, Western blot analysis of FOXO3a and tubulin β expression in total extracts of Hdh cells. *B*, quantification of data in *A*. FOXO3a levels were normalized to tubulin β levels. *C*, Western blot analysis of FOXO3a in nuclear and cytoplasmic fractions of Hdh cells. HDAC2 and tubulin β served as controls for nuclear and cytoplasmic fractions, respectively. *D* and *E*, quantification of FOXO3a levels in nuclear (*D*) and cytoplasmic fractions (*E*) of Hdh cells. For normalization, FOXO3a signal intensities were normalized to HDAC2 or tubulin β signal intensities for nuclear or cytoplasmic fractions, respectively, of each Hdh cell line. The relative FOXO3a levels in Hdh^7/7^ cells were arbitrarily set as 1. The statistical significance shown with *asterisks* is relative to the levels measured from Hdh^7/7^ cells. *, *p* < 0.05; *n* = 4

To compare FOXO3a protein expression levels in Hdh^7/7^, Hdh^7/109^, and Hdh^109/109^ cells, we prepared total cell lysates from all three cell lines and estimated the relative amount of FOXO3a protein by immunoblotting using antibodies against FOXO3a (Fig. 2*A*). FOXO3a-specific signal intensities were quantified densitometrically and normalized to the amount of tubulin β measured from the same extracts. Protein levels of FOXO3a showed significantly more than 2-fold increase in mutant Hdh^7/109^ and Hdh^109/109^ cell lines, respectively, compared with FOXO3a protein level in wild type Hdh^7/7^ cells (Fig. 2*B*; *n* = 4, *p* = 0.035 and *p* = 0.026, respectively).

Elevated levels of FOXO3a protein seen in total lysates and differential immunocytochemical staining in mutant Hdh cells raised the question of distribution of excess FOXO3a in those cells. To answer this, nuclear and cytosolic fractions were prepared, and equal amounts of protein extracts were resolved by SDS-PAGE and analyzed by Western blotting with FOXO3a antibodies (Fig. 2*C*). To assess the quality of fractionation and monitor loading, histone deacetylase 2 (HDAC2) and tubulin β were detected as nuclear and cytosolic markers, correspondingly. Protein levels of FOXO3a were increased ∼2-fold in both nuclear (Fig. 2*D*) and cytosolic fractions (Fig. 2*E*) of Hdh^109/109^ cells compared with WT cells (*n* = 4, *p* = 0.012 and *p* = 0.035, respectively). In Hdh^7/109^ cells, a 1.4-fold borderline increase was detected in both fractions (Fig. 2, *D* and *E*; *n* = 4). Collectively, these results indicate that the overall elevated FOXO3a protein content in mutant Hdh cells is associated with its increased levels both in the cytoplasm and nuclei.

##### Activation of AKT Is Not Reduced in Mutant Hdh Cells

**FIGURE 3.**
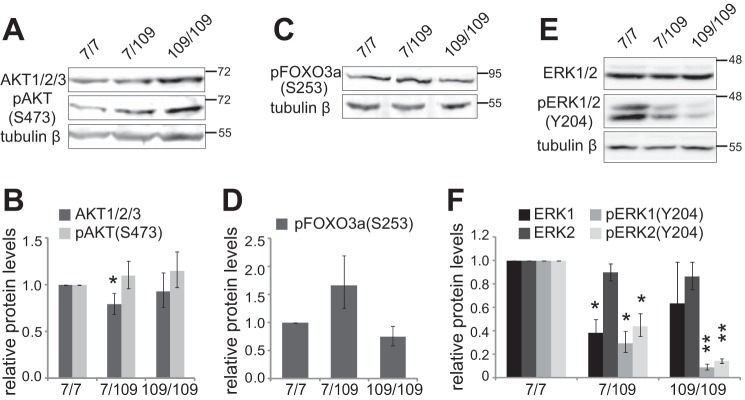
**AKT and ERK activity in Hdh cells.** *A*, Western blot analysis of AKT1/2/3 proteins and AKT1/2/3 phosphorylated at serine 473 (pAKT1/2/3(Ser-473)) in Hdh cells. Tubulin β served a loading control. *B*, quantification of the signals of AKT1/2/3 and pAKT1/2/3(Ser-473) in *A*. Signals were normalized to the levels of tubulin β. *C*, Western blot analysis of FOXO3a phosphorylated at serine 273 (pFOXO3a(Ser-273)) in Hdh cells. Tubulin β levels were determined in control. *D*, quantification of the data in *C*. pFOXO3a(Ser-273) signals were normalized to the levels of tubulin β. *E*, Western blot analysis of ERK1/2 and ERK1/2 phosphorylated at tyrosine 204 (pERK1/2(Tyr-204)) in Hdh cells. Tubulin β was utilized for loading control. *F*, quantification of the signals of both ERK isoforms and phosphorylated isoforms in *E*. Signals were normalized to the levels of tubulin β or HDAC2. The statistical significance shown with *asterisks* is relative to the levels measured from Hdh^7/7^ cells. *, *p* < 0.05; **, *p* < 0.01; ***, *p* < 0.001; *n* = 3

The main kinase responsible for phosphorylation and nuclear export of FOXO3a protein is AKT (20). Because increased nuclear FOXO3a was present in HD cells, we asked whether AKT signaling might be compromised in these cells. First, we determined the levels of total AKT and activated AKT (phosphorylated at Ser-473) in Hdh cells by immunoblotting (Fig. 3*A*). Signal intensities were quantified densitometrically and normalized to the amount of tubulin β. Levels of total AKT did not change dramatically in Hdh cell lines, and a 20% reduction in Hdh^7/109^ cells compared with Hdh^7/7^ cells was observed (Fig. 3*B*; *n* = 4, *p* = 0.045). Protein levels of pAKT1/2/3(Ser-473) in heterozygous and homozygous mutant cells did not differ from the levels in WT cells (Fig. 3*B*; *n* = 3). Second, we measured the levels of FOXO3a phosphorylated at an AKT site in the forkhead domain (Ser-253) in Hdh cells (Fig. 3*C*). Compared with Hdh^7/7^ cells there was a tendency for increased pFOXO3a(S253) levels in Hdh^7/109^ cells and for decreased pFOXO3a(S253) levels in Hdh^109/109^ cells (Fig. 3, *C* and *D*; *n* = 3, 1.7-fold and 0.7-fold, respectively). Altogether, these results indicate that increased nuclear levels of FOXO3a cannot be attributed to reduced AKT signaling in HD cells.

##### Levels of Activated ERK Are Reduced in Mutant Hdh Cells

The ERK kinase phosphorylates FOXO3a initiating its translocation from nucleus to cytoplasm, reducing its transcriptional activity and promoting its degradation (24). Therefore to determine whether ERK might play a role in increased FOXO3a levels in mutant Hdh cells, we measured the levels of total ERK1/2 and phosphorylation-activated ERK1/2 (Tyr-204) in Hdh cells by immunoblotting (Fig. 3*E*). Although ERK1 levels in Hdh^7/109^ cells were reduced to 42% of the levels in WT cells (Fig. 3*F*; *n* = 3, *p* = 0.0088), levels of ERK1 in Hdh^109/109^ cells and ERK2 levels both in Hdh^7/109^ and Hdh^109/109^ cells were not significantly altered compared with Hdh^7/7^ cells (Fig. 3*F*). On the contrary, levels of phosphorylated ERK1/2 were reduced drastically in both mutant huntingtin expressing cell lines (Fig. 3*E*). In Hdh^7/109^ cells, pERK1 levels were 32% and pERK2 levels were 47% of the levels measured in Hdh^7/7^ cells (Fig. 3*F*, *n* = 3, *p* = 0.0026 and *p* = 0.005, respectively). The amount of pERK detected in Hdh^109/109^ cells was further decreased, pERK1 to 12% and pERK2 to 19% of the activated ERK levels in Hdh^7/7^ cells (Fig. 3*F*, *n* = 3, *p* = 0.001 and *p* = 0.0022, respectively). Next we attempted to measure the levels of FOXO3a phosphorylated by ERK kinases at Ser-294 to see whether changes in kinase activity correlate with its target protein condition. Unfortunately, we could not detect any signal of pFOXO3a(Ser-294) in Hdh cells, indicating that the level of phosphorylation of FOXO3a in these cells is below the detection limit of the used antibodies (data not shown). Collectively, the above data show that the levels of ERK kinases phosphorylated at Tyr-204 are significantly lower in HD model cells. Even though we were unable to measure pFOXO3a(Ser-294) levels, it remains possible that the observed increase of FOXO3a in mutant Hdh cells may partly result from inactivation of ERK kinases.

##### Nuclear FOXO3a Levels Are Increased in 3-NP-treated Neurons

**FIGURE 4.**
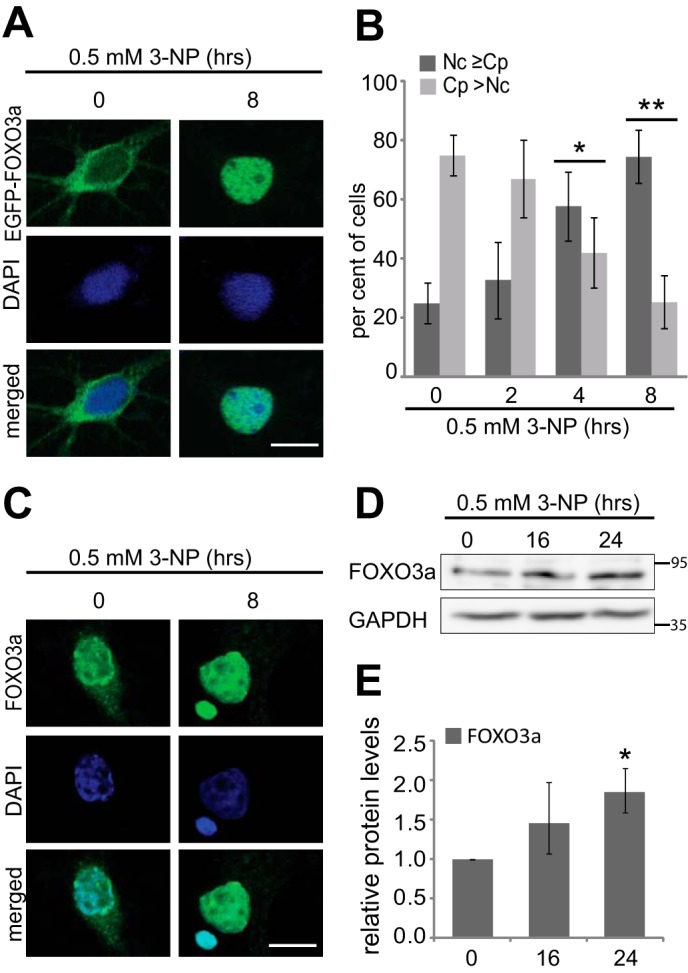
**Effect of mitochondrial toxin 3-NP treatment on subcellular distribution and levels of FOXO3a in cortical neurons.** *A* and *C*, representative confocal microscopy images of overexpressed EGFP-FOXO3a (*A*) or endogenous FOXO3a-like signal (*C*) in cortical neurons left untreated or treated with 3-NP for 8 h. Localization of EGFP-FOXO3a was detected by direct fluorescence, endogenous signal was detected immunocytochemically with FOXO3a antibodies, and DNA was counterstained with DAPI. *Scale bar*, 10 μm. *B*, quantitative analysis showing percentages of primary cortical neurons with mainly nuclear (Nc ≥ Cp) or mainly cytoplasmic (Cp > Nc) EGFP-FOXO3a localization when left untreated (0 h) or treated with 0.5 mm 3-NP for 2, 4, or 8 h. Shown are the mean results from three independent experiments with 35–150 neurons counted in each experiment for each time point. *D*, Western blot analysis of FOXO3a protein levels in cortical neurons left untreated or treated with 0.5 mm 3-NP for 16 or 24 h. GAPDH served as a loading control. *E*, quantification of data in *D*, FOXO3a signals were normalized to the levels of GAPDH. The statistical significance shown with *asterisks* is relative to the levels measured from untreated neurons. *, *p* < 0.05; **, *p* < 0.01; *n* = 3.

The most vulnerable cells in HD are striatal and cortical neurons. To study the distribution of FOXO3a in the disease relevant cell type, we determined the impact of mitochondrial toxin 3-NP on FOXO3a localization in rat primary cortical neurons. Cultured neurons were transfected with EGFP-FOXO3a fusion protein encoding vector and treated with 0.5 mm 3-NP for 2, 4, or 8 h. EGFP-FOXO3a was visualized by confocal microscopy, and the representative images of neurons left untreated and treated with 3-NP for 8 h are shown in Fig. 4*A*. Three independent experiments were performed, and from each indicated time point at least 35 neurons were analyzed and divided into two categories based on whether the EGFP-FOXO3a signal was mainly nuclear (Nc ≥ Cp) or mainly cytoplasmic (Cp > Nc). Time-dependent gradual translocation of EGFP-FOXO3a into the nucleus was observed in accordance to the time of treatment (Fig. 4*B*). 75% of untreated neurons showed cytoplasmic localization of EGFP-FOXO3a. Compared with untreated cells, the number of cells with mainly nuclear EGFP-FOXO3a localization tended to increase after 3-NP treatment for 2 h, whereas in case of longer 3-NP treatments, for 4 and 8 h, mainly nuclear localization was seen in 58 and 75% of neurons, correspondingly (*n* = 3, *p* = 0.32, *p* = 0.012, and *p* = 0.0098, respectively). Subsequently, we analyzed the localization of endogenous FOXO3a protein in control and 3-NP-treated rat primary cortical neurons by immunocytochemical staining with FOXO3a antibodies. Similarly to the results obtained with the overexpressed EGFP-FOXO3a protein, 3-NP treatment induced translocation of endogenous FOXO3a protein into the nucleus (Fig. 4*C*). In untreated neurons, endogenous FOXO3a-like signal was mostly nuclear but was also detected in the cytoplasm, whereas in 3-NP-treated neurons, only nuclear staining was seen.

We observed increased FOXO3a protein levels concurrent with its translocation toward nucleus in mutant Hdh cells. To test whether FOXO3a translocation is accompanied by changes in FOXO3a protein levels in cortical neurons also, we analyzed FOXO3a protein levels by immunoblotting in neurons treated with 3-NP for 16 or 24 h. We found that compared with untreated neurons, FOXO3a levels showed a tendency toward increase at 16 h (1.5-fold, *n* = 3; *p* = 0.17) and were significantly increased at 24 h of treatment (Fig. 4, *D* and *E*; 1.9-fold, *n* = 3; *p* = 0.02). Taken together, these data demonstrate that 3-NP-induced mitochondrial stress leads to nuclear translocation and increased levels of FOXO3a protein in primary cortical neurons.

##### mRNA Levels of Foxo3a and Its Target Gene FasL Are Elevated in HD Cells

**FIGURE 5.**
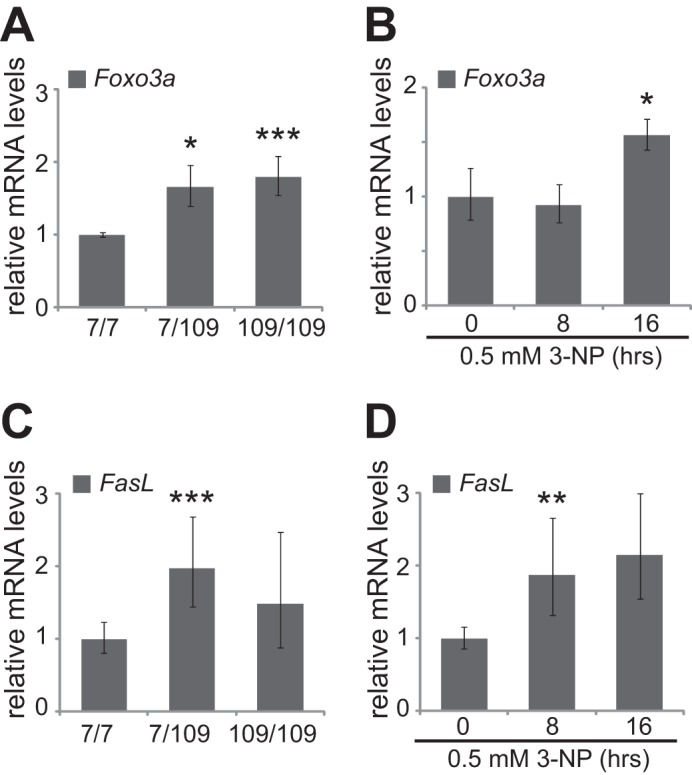
**Foxo3a and *FasL* mRNA levels in HD model cells.** RT-qPCR analysis of *Foxo3a* (*A* and *B*) and *FasL* (*C* and *D*) in Hdh cells (*A* and *C*) and in primary cortical neurons left untreated or treated with 3-NP for 8 or 16 h (*B* and *D*). *Foxo3a* or *FasL* levels were normalized to the levels of *HPRT1*. The mean values and statistical significance shown with *asterisks* are relative to the levels measured in Hdh^7/7^ cells or untreated neurons. *, *p* < 0.05; **, *p* < 0.01; ***, *p* < 0.001; *n* = 4 in *A* and *C*; *n* = 5 in *B* and *D*.

Because FOXO3a nuclear localization was accompanied by increased FOXO3a protein levels in the studied HD model cells, we determined *Foxo3a* mRNA levels in Hdh cells and also in 3-NP-treated primary cortical neurons by RT-qPCR. As demonstrated in Fig. 5*A*, expression of *Foxo3a* was increased 1.7-fold in heterozygous mutant Hdh^7/109^ cells and 1.8-fold in homozygous mutant Hdh^109/109^ cells compared with the expression levels in wild type cells (*n* = 4, *p* = 0.021 and *p* = 0.0049, respectively). Treatment of cortical neurons with 3-NP for 16 h resulted in a 1.6-fold increase in *Foxo3a* mRNA levels compared with untreated neurons (Fig. 5*B*; *n* = 5, *p* = 0.043). Therefore, increased levels of FOXO3a protein in HD cells might be at least partially caused by the up-regulation of *Foxo3a* mRNA expression.

To determine whether increased amounts of nuclear FOXO3a in HD cells have functional consequences, we quantified the mRNA levels of *FasL* (*Fas ligand*), one of the target genes of FOXO3a (20), in Hdh^7/7^, Hdh^7/109^, and Hdh^109/109^ cells, as well as in untreated and 3-NP-treated primary cortical neurons by RT-qPCR. Compared with wild type Hdh^7/7^ cells, the mRNA levels of *FasL* were elevated 2-fold in Hdh^7/109^ cells (Fig. 5*C*; *n* = 4, *p* = 0.0032). Compared with untreated neurons, we observed a statistically significant 1.9-fold increase in *FasL* expression after 8 h of 3-NP treatment and a 2.2-fold increase at 16 h of treatment (Fig. 5*D*; *n* = 5, *p* = 0.0088 and *p* = 0.055, respectively). These data suggest functional consequences of FOXO3a translocation into nucleus on its target genes.

##### FOXO3a mRNA Levels Are Elevated in Brain Tissue of R6/2 Mice and HD Patients

**FIGURE 6.**
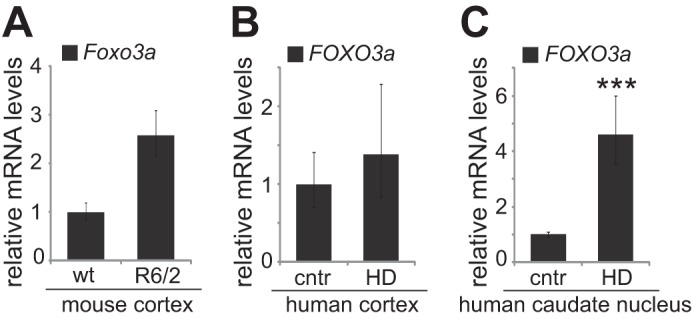
***In vivo* expression levels of *FOXO3a* mRNA.** *A*, RT-qPCR analysis of *Foxo3a* levels in the cerebral cortex of WT and R6/2 6-week-old mice. *HPRT1* levels were determined for normalization. Shown are the mean results from three pairs of mice. *B* and *C*, *FOXO3a* levels in the cerebral cortex (*B*) and caudate nucleus (*C*) of HD patients and nondiseased controls (*cntr*). Total RNAs extracted from post-mortem human brain tissue were reverse transcribed and subjected to qPCR. *FOXO3a* levels were normalized to the levels of *HPRT1*, *SDHA*, and *HMBS*. Shown are the mean results from five (*B*) or three (*C*) patients and nondiseased controls. The statistical significance shown with *asterisks* is relative to the controls. ***, *p* < 0.001

R6/2 transgenic mice carry a fragment of human *HTT* gene with 144 polyglutamine repeats and show fast progress of HD symptoms (34). We analyzed mRNA levels of *Foxo3a* in cortex of R6/2 mice and detected a tendency toward increased expression compared with wild type littermate tissue (Fig. 6*A*; 2.6-fold, *n* = 3, *p* = 0.064). To study *FOXO3a* mRNA levels in HD patients, we performed RT-qPCR of diseased and nondiseased post-mortem cerebral cortex and caudate nucleus tissue. Variable levels of *FOXO3a* mRNA were detected both in HD patient (one grade 2 patient, three grade 3 patients, and one grade 1 patient) and control cortex tissue (Fig. 6*B*; *n* = 5, *p* = 0.26). However, we observed a significant 4.6-fold increase in *FOXO3a* mRNA levels in patient caudate nucleus tissue (two grade 3 patients and one grade 4 patient) compared with controls (Fig. 6*C*; *n* = 3, *p* = 0.00068). These *in vivo* data from R6/2 mice and HD patients further corroborate our findings in cells *in vitro* about dysregulated FOXO3a activity in HD.

##### Transcription Factor FOXO3a Binds to Its Own Promoter

**FIGURE 7.**
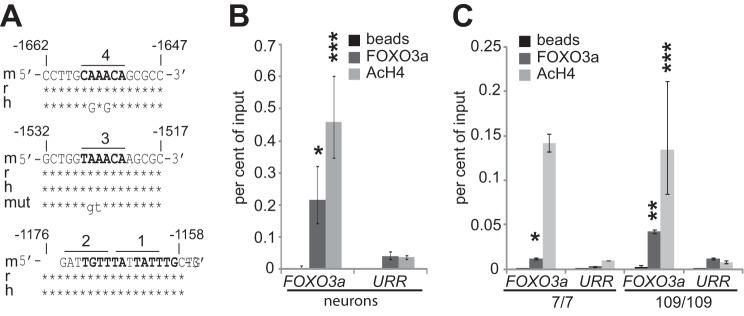
**FOXO3a binds to its own promoter.** *A*, *in silico* analysis of potential FHREs in mouse, rat, and human *FOXO3a* promoter. Shown are the conserved sequences of promoter regions containing the four identified sites that are marked in *bold*. The sequence of mutated FHRE 3 is also shown. The *numbers* indicate the positions relative to the transcription start site. *B* and *C*, chromatin immunoprecipitation analysis demonstrating FOXO3a binding to FHREs containing region in *Foxo3a* promoter in rat primary cortical neurons (*B*) and Hdh^7/7^ and Hdh^109/109^ cells (*C*). Soluble chromatin was co-immunoprecipitated with antibodies specific for FOXO3a or AcH4 or with beads alone. DNA from *Foxo3a* promoter or from an URR was amplified by qPCR. The data are presented as percentages of input DNA. The statistical significance denoted with *asterisks* is relative to values of URR obtained with the respective antibodies. *, *p* < 0.05; **, *p* < 0.01; ***, *p* < 0.001; *n* = 3 (except in case of AcH4 ChIP in Hdh^7/7^ cells, where *n* = 2).

Increased nuclear localization of FOXO3a protein and accompanying higher expression level of *Foxo3a* mRNA in HD cells led us to the hypothesis that positive autoregulation might be involved in FOXO3a signaling. By *in silico* analysis, we identified four potential Forkhead response elements (FHREs), named 1–4, in *Foxo3a* promoter less than 2 kb upstream from the transcription start site (Fig. 7*A*). We carried out chromatin immunoprecipitation experiments with primary neurons and Hdh^7/7^ and Hdh^109/109^ cells using FOXO3a antibodies. Acetyl histone 4 (AcH4) specific antibodies were used as positive control, and beads-only precipitated samples showed a general experimental background. To study FOXO3a binding to its own promoter, we analyzed the precipitated DNA by RT-qPCR with primers specific for the *Foxo3a* promoter region containing the potential FHREs. Amplification with primers designed against an unrelated untranscribed region (URR) on mouse or rat chromosomes (chromosome 10 and 1, respectively) served as negative control indicating random binding of the antibodies used. As shown in Fig. 7*B*, we detected significant enrichment of AcH4 on *Foxo3a* promoter compared with URR in rat primary cortical neurons, indicating active transcription of the gene (*n* = 3, *p* = 0.00057, *p* = 0.0016). Importantly, higher amounts of *Foxo3a* promoter DNA compared with URR were precipitated with FOXO3a antibodies in primary neurons (Fig. 7*B*, *n* = 3, *p* = 0.032). In WT and mutant Hdh cells, similar amounts (∼0.13%) of input DNA were precipitated by AcH4 antibody, whereas 0.01 and 0.04% of input DNA was precipitated by FOXO3a antibodies in Hdh^7/7^ and Hdh^109/109^ cells, respectively (Fig. 7*C*; *n* = 3, *p* = 0.022 and *p* = 0.0076 compared with URR). These results demonstrate binding of endogenous FOXO3a protein to its own promoter in all studied cell types.

##### Regulation of FOXO3a Transcription Factor Involves Positive Feedback

**FIGURE 8.**
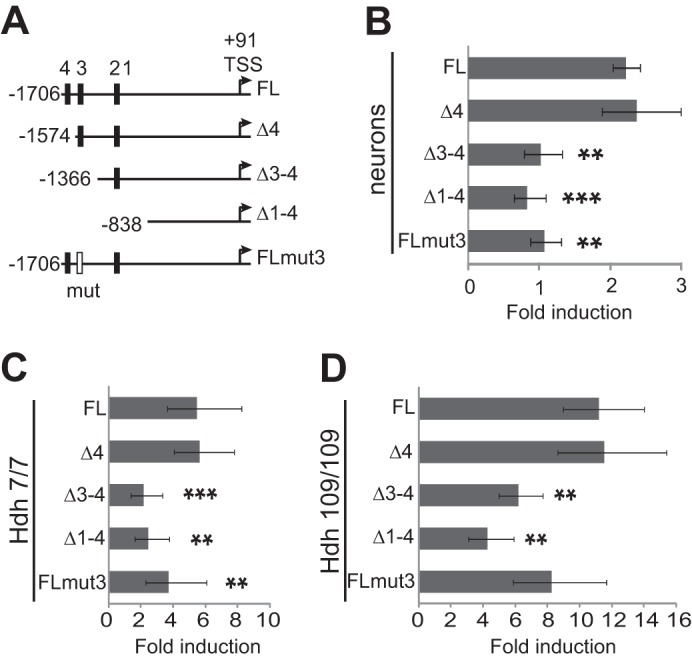
**FOXO3a activates transcription from its own promoter.** *A*, schematic representation of the *Foxo3a* promoter regions cloned into firefly luciferase reporter vector. The locations of potential FHREs 1–4 are indicated with *black boxes*. Mutated FHRE 3 is depicted as a *white box*. The *numbers* indicate the position relative to the transcription start site (*TSS*) that is shown with *arrows. B–D*, reporter assays with primary cortical neurons (*B*), Hdh^7/7^ (*C*), and Hdh^109/109^ cells (*D*) transfected with *Foxo3a* promoter constructs and *Renilla* luciferase construct with *EF1*α promoter (*B*) or *PGK1* promoter (*C* and *D*) for normalization. The cells were co-transfected with constructs encoding constitutively active Flag-FOXO3A-TM or empty vector. Luciferase activities were measured, and data are presented as fold induction of normalized luciferase activity in Flag-FOXO3A-TM-expressing cells compared with cells transfected with empty vector. The statistical significance denoted with *asterisks* is relative to the induction of transcription from WT full-length promoter. *, *p* < 0.05; **, *p* < 0.01; ***, *p* < 0.001; *n* > 3

To elucidate which of the potential FHREs are responsible for FOXO3a binding to its own promoter, we cloned mouse *Foxo3a* promoter regions of different lengths into a *luc2P* reporter vector (Fig. 8*A*) and assessed the ability of Flag-tagged constitutively active triple mutant (TM) of FOXO3a (T32A,S253A,S315A-mutated AKT kinase phosphorylation sites) (20) to activate transcription from these promoters in rat primary cortical neurons and Hdh cell lines. Normalized activity of reporter transcribed from *Foxo3a* promoter encompassing potential FHRE sites 1–4 was increased more than 2-fold in neurons, more than 5-fold in Hdh^7/7^ cells and more than 11-fold in Hdh^109/109^ cells expressing Flag-FOXO3a-TM compared with cells transfected with empty vector (*n* = 4, *p* = 0.00051, *p* = 0.0056, *p* = 0.0027, respectively; Fig. 8, *B–D*). Deletion of potential FHRE site 4 did not affect induction of promoter activation by Flag-FOXO3a-TM in any of the cell types (Fig. 8, *B–D*). However, induction of transcription from *Foxo3a* promoters containing potential FHRE sites 1–2 only or none of the FHREs was significantly decreased compared with the induction of the longest promoter in all of the cell types (Fig. 8, *B–D*). These results suggest that site 3 FHRE (TAAACA) is to a large extent responsible for the transcriptional autoregulation of FOXO3a. To verify this, we used site-directed mutagenesis to introduce two point mutations into the site 3 FHRE (Fig. 7*A*) in the context of the longest cloned *Foxo3a* promoter (Fig. 8*A*) and analyzed the effect of the mutation on the ability of Flag-FOXO3a-TM to activate reporter transcription. As shown in Fig. 8*B*, Flag-FOXO3a-TM was not able to activate transcription from *Foxo3a* promoter carrying mutations in site 3 FHRE in neurons, indicating that this site was indeed needed for autoregulation in these cells (*n* = 4, *p* = 0.0011 compared with WT promoter). Activation of transcription from the mutant *Foxo3a* promoter compared with WT full-length promoter was also reduced in Hdh^7/7^ cells (*p* = 0.0068; Fig. 8*C*), whereas the decrease seen in Hdh^109/109^ cells remained statistically nonsignificant (*p* = 0.063; Fig. 8*D*). Compared with neurons, the induction of reporter transcription from *Foxo3a* promoters without site 3 FHRE or with mutant site 3 FHRE were higher in Hdh cells, especially in Hdh^109/109^ cells, indicating that other binding sites may also play a role. Altogether, the above findings show that transcription factor FOXO3a is able to activate its own transcription by directly binding to its own promoter.

## DISCUSSION

FOXO transcription factors have been implicated in neurodegenerative diseases, but their exact roles in these diseases have remained controversial (35–41). Here we report differential immunocytochemical staining with FOXO3a antibodies in striatal Hdh cells expressing wild type and/or mutant huntingtin endogenously. We detected elevated FOXO3a protein levels in the nuclei of mutant Hdh cells and 3-NP-treated rat primary cortical neurons. Additionally, our results show that *FOXO3a* mRNA expression levels are increased in mutant Hdh cells, in 3-NP-treated primary neurons and in post-mortem caudate tissue of HD patients. Moreover, using chromatin immunoprecipitation and reporter assays, we demonstrate that FOXO3a directly regulates its own expression, thus forming a positive autoregulation loop.

Our subcellular fractionation experiments showed elevated FOXO3a protein levels both in the cytoplasm and nucleus in Hdh^109/109^ cells compared with Hdh^7/7^ cells. The elevated nuclear import of FOXO3a in mutant Hdh cells might be due to increased cellular stress and/or decreased growth factor signaling that are known to be caused by mutant huntingtin (42, 43). One of the kinases activated by growth factor signaling is AKT that plays a central role in promoting the survival of a wide range of cell types, partly through inactivation of FOXO factors (20, 44, 45). Controversial results describing AKT activity in HD have been reported earlier. Increased levels of activated AKT were detected in striata of Hdh^111/111^ knock-in mice and cultured mutant Hdh^111/111^ striatal cells, compared with their wild type counterparts, whereas no changes in phosphorylated FOXO1 levels were detected (46). In another study, both activated and total AKT levels were shown to be reduced in rat HD models and in lymphoblasts, lymphocytes, and post-mortem brain extracts from HD patients (47). Additionally, unchanged AKT activation in response to BDNF treatment has been reported in Hdh cells (9). Here we showed that AKT activity is not compromised in mutant Hdh cells. According to our results, total AKT levels were not reduced in Hdh^109/109^ cells compared with WT cells, and no significant differences in pAKT1/2/3(S473) or pFOXO3a(S253) levels were detected in Hdh cells with different genotypes. Therefore, increased FOXO3a nuclear levels in mutant Hdh cells seen in this study cannot be attributed to disturbed AKT activation *per se*. One explanation for increased levels of nuclear FOXO3a in HD cells could be that the amount and activity of AKT is not sufficient to maintain low levels of nuclear FOXO3a in the presence of increased amounts of the transcription factor.

Another possibility is that other signaling pathways, which modulate FOXO3a activity, are altered in mutant Hdh cells. Here we demonstrated that phosphorylated and activated ERK kinase levels are significantly lower in mutant Hdh cells. ERK has been shown to phosphorylate FOXO3a at three serine residues (Ser-294, Ser-344, and Ser-425), promoting its translocation into cytoplasm (24). Furthermore, ERK-phosphorylated FOXO3a is substrate for E3 ligase MDM2 and is directed to proteasomal degradation (24). In general, ERK1/2 is activated by oxidative stress for neuroprotection, but in Hdh^111/111^ cells, unlike in Hdh^7/7^ cells, H_2_O_2_ treatment does not induce ERK pathway, and these cells do not respond to protective BDNF treatment either (9). In contrast, for PC12 and ST14A cells, it has been shown that expression of mutant Htt activates ERK and inhibition of apoptotic caspases is seen (48). Although we were not able to detect pFOXO3a(S294) in Hdh cells, it remains possible that FOXO3a phosphorylation at this or other ERK target sites might be compromised in mutant Hdh cells. Therefore, the increased level of FOXO3a and its nuclear location in Hdh^7/109^ and Hdh^109/109^ cells might partly result from decreased phosphorylation of FOXO3a by ERK kinases.

In addition, activity of JNKs, which facilitate FOXO nuclear import, has been shown to be activated by oxidative stress (37) and growth factor deprivation (49) and to be increased in a rat HD model (50). So far, phosphorylation by JNK has only been shown for FOXO4 and other FOXO family proteins lack the region targeted by JNK in FOXO4 (51). Another up-regulated kinase in HD is 5′ AMP-activated protein kinase (AMPK), which is activated in response to bioenergetic failure induced by excitotoxic injury (52). AMPK acts in two ways on FOXO3a: first it blocks AKT activation and prevents FOXO3a phosphorylation by AKT, and second, AMPK phosphorylates FOXO3a in the nucleus increasing its binding to target gene *Bim* (36). Hence, it would be of interest to further study JNK and AMPK and related kinases activity in conjunction with phosphorylation of FOXO3a in HD.

A chemical model of HD utilizing mitochondrial toxin 3-NP has been widely used in rodents and also in non-human primates to mimic pathological neurodegeneration seen in HD in humans (10, 11, 33). Our results demonstrated that in Hdh^7/7^ cells, 3-NP treatment leads to similar FOXO3a localization change toward the nucleus as was seen in genetic HD models, Hdh^7/109^ and Hdh^109/109^ cells. Also, 3-NP treatment of rat primary cortical neurons led to increased nuclear translocation of endogenous FOXO3a and overexpressed EGFP-FOXO3a. Although endogenous FOXO3a-like signal showed differential initial distribution compared with EGFP-FOXO3a signal in neurons, both were located exclusively to the nuclei of primary cortical neurons after 3-NP treatment. This seeming discrepancy in subcellular localizations of endogenous and exogenous FOXO3a might arise from very different expression levels of the proteins. It is possible that the nucleus in EGFP-FOXO3a transfected neurons is saturated, and excessive protein is transported to cytoplasm; a similar phenomenon has been described for a GFP fusion protein previously (53). Additionally, in our experiments 3-NP treatment of primary neurons induced similar elevation of endogenous FOXO3a protein levels as observed in genetic HD model. Although stresses caused by mutant huntingtin protein and 3-NP induced comparable subcellular localization and protein level changes of FOXO3a, it cannot be excluded that the underlying mechanisms might be different, just as described previously for the causes of cellular energy collapse induced by mHtt or 3-NP (54).

FOXO3a translocation into the nucleus enables FOXO3a to activate its target genes. Here we show that FOXO3a target gene *FasL* is up-regulated in mutant Hdh cells and 3-NP-treated cortical neurons compared with WT Hdh cells and untreated neurons, respectively. In previous studies, controversial results considering FASL expression have been obtained with HD model cells and patient brain samples. Experiments with rat striata-derived cells expressing different N-terminal huntingtin fragments showed heightened *FasL* levels (55), whereas decreased FASL protein levels in the caudate and putamen but not in the parietal cortex of post-mortem human HD brain have been reported (56). These differences could reflect different disease stages.

Our results reveal that in addition to *FasL*, *Foxo3a* mRNA levels are elevated in mutant Hdh cells and 3-NP-treated cortical neurons and most importantly in HD patient post-mortem caudate nucleus. Additionally, we show by chromatin immunoprecipitation and reporter assays that FOXO3a binds to and activates transcription from its own promoter, thus forming a positive feedback loop. Deletion and mutation analysis of *Foxo3a* promoter reporter constructs demonstrated that the principal FHRE required for FOXO3a protein to activate transcription from its own promoter is site 3, with perfect consensus sequence for FOXO3a binding. Although, comparison of our results from reporter assays with cortical neurons, WT and mutant Hdh cells reveals that the importance of different FHREs might vary slightly in different cell types and/or in response to mHtt expression. Previous studies have demonstrated the capacity of FOXO factors to directly activate *Foxo1* and *Foxo4* transcription (57, 58), and during the preparation of this manuscript, a paper was published by Lützner *et al.* (59), describing the ability of FOXO3a to bind different FHREs in *Foxo3a* promoter by electrophoretic mobility shift assay and to up-regulate its own promoter in reporter assays. The latter work is in agreement with ours, suggesting positive feedback mechanism in the regulation of *Foxo3a* expression. Of the potential FHREs analyzed by Lützner *et al. in vitro*, the highest affinity of FOXO3a was shown for site 1–2 FHREs (according to our numbering), but the functionality of these sites in cells was not demonstrated. Importantly, the site 3 FHRE, identified to be largely responsible for autoregulation in this study, was also bound by FOXO3a *in vitro* (59). We found that compared with *FasL*, the dynamics of *FoxO3a* regulation in response to 3-NP treatment in neurons differed. The levels of *FasL* mRNA increased earlier (at the 8-h time point), possibly because of nuclear translocation of already existing FOXO3a protein. The up-regulation of *FoxO3a* transcription followed later (at the 16-h time point), presumably requiring additional factors. Although the increased *FOXO3a* mRNA expression demonstrated in the current study could at least partially explain its higher protein levels in HD cells, it remains possible that differential regulation of FOXO3a relative half-life/degradation in the control and mutant Htt or 3-NP stressed cells is also part of the cause, especially because we demonstrated decreased levels of pERK, which has been shown to phosphorylate FOXO3a, promoting its degradation (24). Additionally, proteasomal degradation of many proteins has been shown to be interrupted by mutant HTT, although *in vivo* studies have given controversial results (60). In conclusion, the results presented here, as well as in previous studies, suggest that positive autoregulatory feedback loop helps to sustain the FOXO3a stress response and might be characteristic for FOXO family members in general.

In all of our Huntington disease models, mutant Hdh cells and 3-NP-treated primary neurons, we observed 1.5–2.5-fold elevated FOXO3a protein and/or mRNA levels. In R6/2 mice cortex, a tendency for increased expression was detected. Recent results, on the other hand suggested a ∼60% reduction in FOXO3a levels in the striatum of 14-week-old HTT N171–83Q transgenic mice and a ∼15% decrease in Hdh^111/111^ cells (39). The reasons for these discrepant results remain unknown. However, we confirmed the specificity of the FOXO3a antibodies used here by RNAi-mediated knockdown of FOXO3a expression. Moreover, we found *FOXO3a* mRNA levels to be elevated more than 4-fold over control levels in HD patient caudate, further corroborating our results obtained in HD models.

Elevated FOXO3a activity in HD cells could lead to different outcomes: trigger cell death or promote survival by inducing stress resistance, and the result seems to depend on accompanying signals and factors (61). Previously, apoptosis has been described to be the primary cellular outcome of FOXO3a activation in neurons during growth factor deprivation or increased oxidative stress (62, 63). Given that BDNF levels were decreased and oxidative stress level was raised in HD (6, 43), it is possible that the increased FOXO3a levels observed in this study promote neuron death. Additionally, we detected up-regulated *FasL* expression along with risen FOXO3a, and previously, these changes were shown to lead to FASL-mediated apoptosis in motoneurons (64). Moreover, extrasynaptic *N*-methyl-d-aspartate receptor expression and signaling has been shown to be increased in medium spiny striatal neurons in HD, and nuclear translocation of FOXO3a has been suggested to contribute to *N*-methyl-d-aspartate receptor-dependent neuronal death (40, 65).

In contrast to the above, several lines of evidence support the protective role of FOXO factors in neurodegerative disorders. For instance, FOXO3a and its *Caenorhabditis elegans* homologue daf-16 have been shown to at least partially mediate the protective effect of sirtuins SIRT1 or sir-2.1 against mutant HTT toxicity in Hdh^111/111^ cells or in a nematode HD model, respectively (39, 66). However, the relationship might be more complex, because a recent study demonstrated that activation of SIRT1 and FOXO3a upon glucose deprivation in PC12 cells leads to differential outcomes depending on NGF availability (67). In addition, although WT FOXO3a overexpression and constantly active FOXO3a precipitate the loss of dopaminergic nigral neurons in acute oxidative stress conditions, it has been shown that FOXO3a is essential for reactive oxygen species detoxification in these neurons chronically exposed to chronic mild oxidative stress (41). Furthermore, it was demonstrated recently by Tourette *et al.* (68) that FOXO3a is an essential factor for survival of Hdh^109/109^ cells in stress conditions. Tourette *et al.* evaluated cell mortality in Hdh^7/7^ and Hdh^109/109^ cells in reduced serum condition along with decreased or increased FOXO3a levels. Although in Hdh^7/7^ cells changes in FOXO3a were not detrimental, in Hdh^109/109^ cells silencing FOXO3a significantly increased and overexpression of FOXO3a considerably decreased cell mortality (68). In conclusion, the results of the current study indicate that FOXO3a levels are risen in HD because of overactivated positive autoregulation loop, but further studies are needed to clarify whether FOXO3a activation is neuroprotective or detrimental in different stages of the disease.

## Footnotes

2 The abbreviations used are:

HD: Huntington disease
3-NP: 3-nitropropionic acid
BDNF: brain-derived neurotrophic factor
qPCR: quantitative PCR
HDAC: histone deacetylase
FHRE: Forkhead response elements
AcH: acetyl histone
URR: unrelated untranscribed region
TM: triple mutant
AMPK: AMP-activated protein kinase.
